# Impacts on water quality before and after water line cleaning and disinfection with peracetic acid in swine farms

**DOI:** 10.1093/tas/txag110

**Published:** 2026-07-21

**Authors:** Gabrielle E Doughan, Becca K Walthart, Meredith B Petersen, Cora E Schau, Kristin J Skoland, Justin T Brown, Scott L Radke, Jessica L Bonnema, Danyang Zhang, Chong Wang, Locke A Karriker

**Affiliations:** Department of Veterinary Diagnostic and Production Animal Medicine, Swine Medicine Education Center, Iowa State University College of Veterinary Medicine, Ames, IA 50011, United States; Department of Veterinary Diagnostic and Production Animal Medicine, Swine Medicine Education Center, Iowa State University College of Veterinary Medicine, Ames, IA 50011, United States; Department of Veterinary Diagnostic and Production Animal Medicine, Swine Medicine Education Center, Iowa State University College of Veterinary Medicine, Ames, IA 50011, United States; Department of Veterinary Diagnostic and Production Animal Medicine, Swine Medicine Education Center, Iowa State University College of Veterinary Medicine, Ames, IA 50011, United States; Department of Veterinary Diagnostic and Production Animal Medicine, Swine Medicine Education Center, Iowa State University College of Veterinary Medicine, Ames, IA 50011, United States; Department of Veterinary Diagnostic and Production Animal Medicine, Swine Medicine Education Center, Iowa State University College of Veterinary Medicine, Ames, IA 50011, United States; Department of Veterinary Diagnostic and Production Animal Medicine, Veterinary Diagnostic Laboratory, Iowa State University College of Veterinary Medicine, Ames, IA 50011, United States; Department of Veterinary Diagnostic and Production Animal Medicine, Swine Medicine Education Center, Iowa State University College of Veterinary Medicine, Ames, IA 50011, United States; Department of Statistics, Iowa State University, Ames, IA 50011, United States; Department of Veterinary Diagnostic and Production Animal Medicine, Iowa State University College of Veterinary Medicine, Ames, IA 50011, United States; Department of Statistics, Iowa State University, Ames, IA 50011, United States; Department of Veterinary Diagnostic and Production Animal Medicine, Iowa State University College of Veterinary Medicine, Ames, IA 50011, United States; Department of Veterinary Diagnostic and Production Animal Medicine, Swine Medicine Education Center, Iowa State University College of Veterinary Medicine, Ames, IA 50011, United States

**Keywords:** biofilm, coliforms, swine, total dissolved solids, water quality

## Abstract

While it is well recognized that maintaining good water quality is a cornerstone of swine husbandry, research on its role in swine health, performance, and management remains limited. This study evaluated the impacts on water quality after a 0.78% peracetic acid administration in water lines for 24 h between groups of pigs to remove water line mineral scale and biofilm. A secondary objective of the study was to evaluate if there were water quality differences between the water sample location (well source vs. rooms). Six, wean-to-finish swine farms in Iowa sourcing water from on-site wells were enrolled in the study and water samples were collected pre-treatment (0), post-treatment (1), and 3, 5, 7, 14, 21, 42, 56, and 77 d post-treatment. Water samples were tested for minerals, nitrate, sulfate, total dissolved solids, on-farm temperature, on-farm and in-lab pH, total coliforms and *E. coli*. Study results demonstrated that most chemical water quality parameters (calcium, copper, iron, magnesium, manganese, molybdenum, phosphorus, potassium, selenium) were not significantly different over sample collection dates, sample locations, and the interaction terms. Total coliforms, total dissolved solids, zinc, on-farm and in-lab pH, and water temperature displayed significant differences. This study showed that total coliforms were not statistically significant pre-and post-treatment with a one-time application of 0.78% peracetic acid for 24 h, and that total coliforms numerically peaked and reached the upper reporting limit at 7 d post-treatment in rooms. Total coliforms were significantly different between sample locations (well source vs. room water lines), with room water lines having greater total coliform counts. These results demonstrate that an application of 0.78% of peracetic acid does not significantly impact most chemical water quality parameters post-treatment. Water sample location demonstrated significant differences in microbial water quality, with rooms water samples having significantly greater total coliforms than well collected samples. This demonstrates that water line biofilms are potentially contributing to decreased water quality in farms. Room vs. well differences were appreciated for total dissolved solids, on-farm and in-lab pH, and water temperature. This research provides further water sampling guidance considering multiple locations and provides additional insights into water line management strategies in swine farms.

## Graphical Abstract

**Graphical Abstract:**
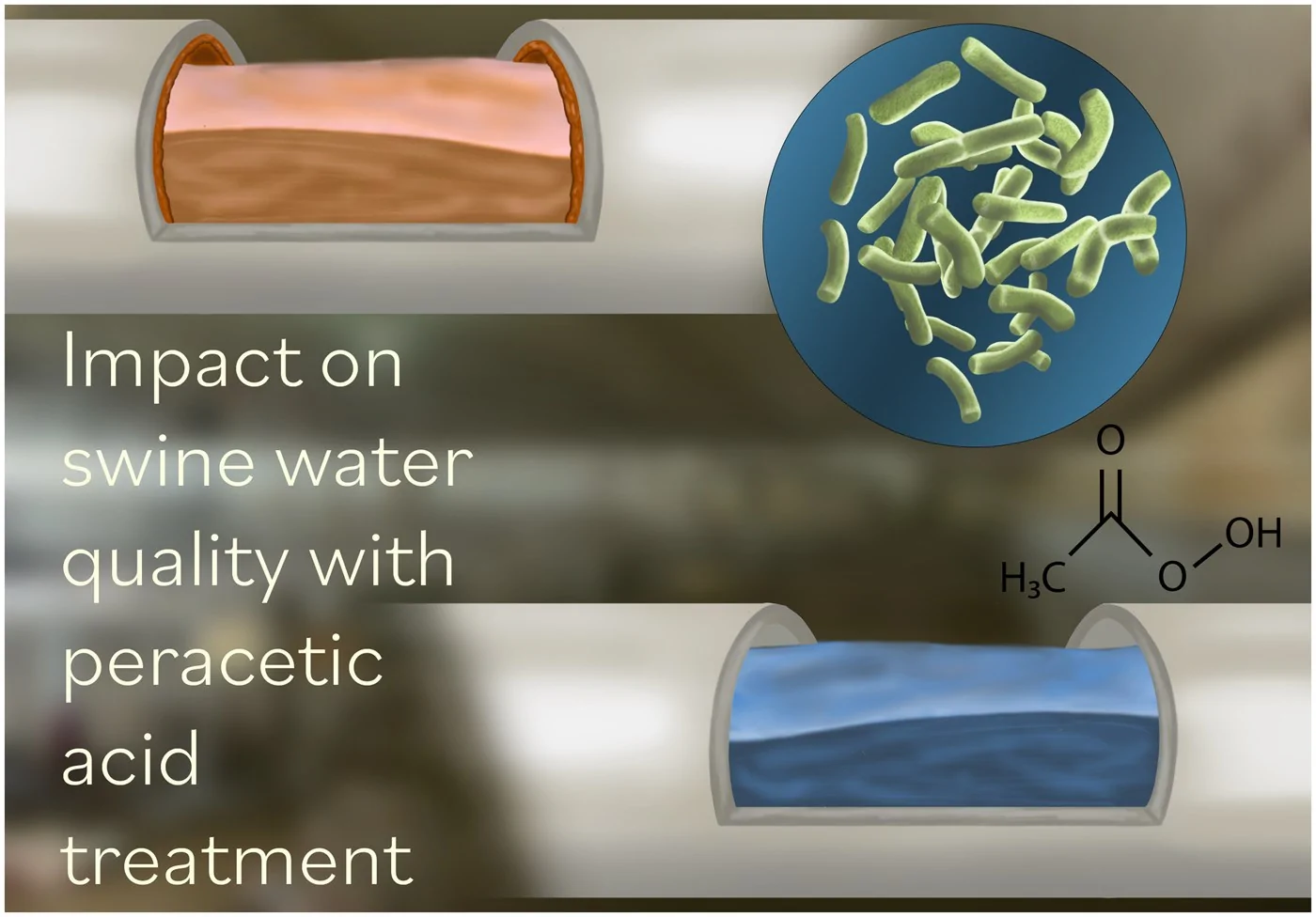

## Teaser

This study evaluated water quality at multiple sampling locations before and after water line cleaning with 0.78% peracetic acid. Results demonstrate that peracetic acid did not significantly improve most water quality analytes, however, room water samples significantly had greater coliform values than wells, potentially associated with biofilm regrowth and shedding.

## Introduction

Water is recognized as a critical nutrient and resource in swine production, influencing feed intake (Edwards and Crabb 2021), thermoregulation, medication administration, and biosecurity. Despite this importance, water quality, ecology, impacts on health and production, and overall management requirements on the farm remain poorly understood. Water quality is evaluated in three major categories, physical properties (turbidity, color, and odor), chemical composition, and microbiological components (Patience 2011; NRC 2012; Edwards and Crabb 2021). Prior research on swine water quality has fixated on the chemical aspects of water quality, ie minerals, pH, total dissolved solids, nitrates, sulfates, etc., and their impact on performance. Water quality standards for swine were established in the late 1970s to early 2000s and relevance to the modern pig is debatable (USEPA 1973; NRC 1974; Toft et al. 1987; Ensley 1998; Socha et al. 2003; Nyachoti et al. 2005; Edwards 2018). Most of this previous work often focused on one water quality parameter in isolation, without assessing other influential factors or considering additive effects of multiple parameter changes (Anderson and Stothers 1978; Paterson et al. 1979; Toft et al. 1987; McLeese et al. 1992; Veenhuizen et al. 1992; Anderson et al. 1994; Carson 2000; Patience et al. 2004; Lozinski et al. 2022). More recently, water quality and its impact on health and performance have been re-assessed for health and production impacts (Lozinski 2020; Lozinski et al. 2022; Samuel 2022), and guidelines for updated swine water quality have also been proposed or reconsidered (Edwards 2018). This body of work has demonstrated that pigs can generally tolerate a wide range of water qualities with minimal to no impact on performance (Nyachoti and Kiarie 2010). Some water quality parameters, such as elevated total dissolved solids (TDS) and sulfates, can cause osmotic diarrhea but have minimal impact on growth performance and overall pig health (Paterson et al. 1979; Ensley 1998; Carson 2000; NRC 2012; Samuel 2022). Younger or health challenged pigs may be more susceptible to water of poor quality (Lozinski et al. 2022).

Although the chemical aspects of water quality for pigs have been assessed, there have been fewer studies evaluating the impact of the microbiological component of water on pig health and production (DeWitt et al. 1987; Ensley 1998; Stull et al. 1999). Microbiological components of water are also subject to contamination depending on how and where the water sample was collected (such as from an un-sanitized water nipple) and results are often interpreted with this consideration. In the literature, a range of water microbial tolerance guidelines have been suggested, but many guidelines are extrapolated from human and other livestock species standards (USEPA 1973; Ensley 1998; Carson 2000; Edwards 2018; Menegat et al. 2019). Microbial contamination of water has been traditionally assessed through the measurement of indicator bacteria such as coliforms (including fecal coliforms) and *Escherichia coli* (*E. coli*). Coliforms are gram-negative bacteria belonging to the *Enterobacteriaceae* family (Paruch and Mæhlum 2012). They are rod-shaped, non-spore-forming, oxidase-negative organisms that ferment lactose and possess the enzyme *β-galactosidase*. Coliforms are also characterized by their growth, growing in aerobic conditions and can demonstrate facultative anaerobic growth at 37 °C. Coliforms have served as indicators of fecal contamination and potential presence of pathogens since the late nineteenth century (Dutka 1973; Tambi et al. 2023). Total coliforms and *E. coli* were selected as indicators as they are representative of characteristics found in enteric organisms of warm-blooded animals (Kilb et al. 2003; Paruch and Mæhlum 2012). However, these organisms can also be present naturally in the environment (Paruch and Mæhlum 2012). There is conflicting evidence of the validity and repeatability of coliforms predicting the presence of other pathogens within a sample (Kilb et al. 2003; Richiardi et al. 2023; Tambi et al. 2023). Water may contain and maintain the viability of other microbials such as viruses, protozoa, fungus, and algae. These organisms could have health effects on pigs, but monitoring mechanisms for these organisms are expensive and not widely available. Therefore, their frequency of occurrence in pig farms is understudied (Doughan et al. 2022, 2023). Regardless, microbial components of water are often the primary complaint of water quality concerns on farms and present a potential biosecurity risk (Nyachoti et al. 2005; Patience 2011; Dewulf et al. 2018; Böger et al. 2020; Olsen and Steven 2020; Edwards and Crabb 2021; Doughan et al. 2022, 2023).

Microbial contamination can originate in swine water systems from two sources, the water source and the potential entry points into the water system (Doughan et al. 2022, 2023). Common water sources for swine include untreated surface water and untreated groundwater (Nyachoti et al. 2005; Nyachoti and Kiarie 2010; Walthart 2024), which are more susceptible to contamination than municipal water supplies that are treated, strictly regulated, and monitored (McAllister and Topp 2012; Olsen and Steven 2020; Doughan et al. 2023). Swine drinking water systems can contribute to further microbial contamination due to biofilm formation in water line pipes supplying the farm (Doughan et al. 2026). Factors such as structural design, internal barn temperature, baseline water quality chemical parameters, and the ability to medicate the water system with antibiotics, probiotics, electrolytes and live attenuated oral vaccines can serve as contributing factors to biofilm development (Little et al. 2021; Doughan et al. 2022, 2023). Hard water (elevations in calcium and magnesium concentrations) and elevated iron concentrations, paired with a basic pH can drive mineral deposits on the insides of water line pipes, decreasing internal diameter size and decreasing water availability to pigs. Biofilms can utilize these mineral formations as a nutrient source and scaffolding to attach and aid in more rapid biofilm development (Fish et al. 2016; Doughan et al. 2022, 2023). However, these interactions in swine water line ecology are poorly understood. Biofilms can lead to the degradation of water quality through dispersal events into the bulk water supply. In human water distribution systems, it has been estimated that 5% of biofilm is released into the water supply, with 95% of the biofilm attached to the interior pipe walls, suggesting that a similar risk may exist in swine facilities (Flemming et al. 2002). Microbial components of water quality and their impacts on pig health and performance are poorly understood and may be a missing component to a comprehensive assessment of optimum water quality for pigs.

Water line cleaning and disinfection may serve as a potential solution to improving water quality in swine facilities. Peracetic acid (PAA), a mixture of hydrogen peroxide and peroxyacetic acid, has been utilized for water line cleaning and disinfection and for water treatment in livestock. Peracetic acid contains hydrogen peroxide, acetic acid and peracetic acid making it a strong oxidizer and acidifier which allow for the removal of mineral scale and biofilms with broad-spectrum antimicrobial activity (Kitis 2004; Farjami et al. 2022; Kampf 2024). Removal of mineral scale and biofilms may improve chemical and microbiological components of water quality for pig drinking water.

The primary objective of this study was to assess components of water quality in swine water systems before and after a one-time water line cleaning and disinfection with a commercial PAA product, CID 2000 Pro (CID LINES, an Ecolab Company, Ghent, Belgium) at a concentration of 0.78% in between groups of pigs in six wean-to-finish farms in Iowa. A second objective was to detect if there were differences in water quality parameters based on the location of the water sample collection (well vs. inside the farm). The null hypothesis was that there is no significant difference in water quality parameters or in the location the water sample was taken before and after a single water line cleaning and disinfection event with 0.78% PAA in six wean-to-finish farms between groups of pigs.

## Materials and methods

### Site criteria and study design

This research was performed through one central sampling population as outlined by Doughan (2025). Commercial, wean-to-finish swine farm sites (*n* = 6) containing two rooms with well water sources in west-central Iowa were enrolled into the observational, longitudinal study. As a function of the study, water and water line samples were collected across 10 time points; pre-treatment (0) with 0.78% PAA, post-treatment (1) (24-h after 0.78% PAA contact time in water lines and after product was flushed from water lines), and 3, 5, 7, 14, 21, 42, 56, and 77 d post-treatment. Samples were collected from May to November in 2023. Detailed methods of the study design, sample collection timeline, and 0.78% PAA water line administration are described elsewhere (Doughan 2025; Doughan et al. 2026). Water consumption, environmental barn temperatures, and well construction information could not be obtained or were not recorded at all sites and were not evaluated in this study. Antimicrobials and electrolytes were allowed to be administered into the water lines, if warranted or as a part of an established protocol, throughout the study. Methods for specific objectives regarding the evaluation of water quality are provided in this manuscript.

### Water sample collection

Water samples were collected from three locations at each sample collection time point: pre-treatment (0), post-treatment (1), 3, 5, 7, 14, 21, 42, 56, and 77 d post-treatment. Source water samples were collected at a well access point as close to the well entrance point in the farm. Water was also collected in the two rooms on the farm at a distal flush valve from a “coupon side stream,” a mechanism described by Doughan (2025) and Doughan et al. (2026). For each water sample, new gloves were donned and the water was allowed to flow for 20 s. After 20 s, a 50 mL falcon tube was filled with sampled water and tested for on-farm pH and temperature. On-farm pH was measured by a handheld digital pH meter (Roulon, Amazon, Washington, USA). The water was then tested for total oxidizing oxygen (TOO) (Doughan 2025) to ensure no active cleaning or disinfection oxidation product was running at the time of sample collection. Once the on-farm pH, temperature, and a TOO test (Ecolab Inc., St. Paul, MN) were obtained and performed, with no evidence of an oxidizing agent present (TOO results), the 50 mL sample was discarded. The sample tap was disinfected by wiping 70% isopropyl alcohol wipes (Medi-First Alcohol Prep-Pad Wipes) until the spigot was clean. A sterile 500 mL bottle (Thermo Scientific Nalgene) was opened and water from the sampling location was collected. The same process occurred for the water samples in the rooms, however instead of a spigot, the flush valve at the distal coupon side stream within the room was cleaned with 70% isopropyl alcohol wipes before the 500 mL sample collection. Information on water administered medications and treatments (electrolytes, probiotics, acidifiers, etc.) were collected from the farm’s records at the time of sample collection.

### Water testing

Water samples were placed on ice and submitted to the Iowa State University Veterinary Diagnostic Lab (ISU VDL) for a mineral panel, water quality (nitrates, sulfates, TDS), total coliform and *E. coli* counts (Colilert, IDEXX Laboratories, Westbrook, Maine, USA), and in-lab pH within 3 d of sample collection. Mineral and water quality parameter concentrations were evaluated via high-performance liquid chromatography, inductively coupled plasma optical emission spectrometry (PlasmaQuant MS Elite Single Quadrupole ICP-MS System, Analytikjena, Jena, Germany) and gravimetric analysis. Mineral concentrations that were evaluated included: calcium, copper, iron, potassium, magnesium, manganese, molybdenum, phosphorus, selenium, and zinc. Sodium was not assessed directly as it is measured as a component of TDS.

Twelve water samples from four sample collection dates could not be submitted to the ISU VDL within 3 d of collection. The samples were frozen at −70 °C on the same day they were collected and thawed before they were submitted to the ISU VDL.

### Total Oxidizing Oxygen (TOO) test

Methods for the TOO test have been reported elsewhere (Doughan 2025). The TOO test was performed to ensure no oxidation product was being run in the water lines at the time of water sample collection.

### Statistical analysis

Water quality parameter averages on all sampled farms from all sampling locations were reported to provide context for the variability in water samples across farms. Water hardness, reported as CaCO_3_, was calculated by taking the ppm values of calcium and magnesium and using the formula (Boyd et al. 2016): {[Calcium (ppm) × 2.497] + [Magnesium (ppm) × 4.118]}. Many water quality parameters were limited to levels of quantification above zero. If a value could not be obtained from this cutoff, it was assumed as zero to create averages for water qualities collected from all farms and sampling locations. Maximum levels of detection were reported in the data for total coliforms and *E. coli* (eg >2419.6001 count per 100 mL). For example, samples that reported > 2419.6001 for coliforms or *E. coli* were reported at 2419.6001 to calculate the average and ranges from all farms and sampling locations.

Statistical analysis on water quality parameters was carried out using Statistical Analysis System (SAS, version 9.4, SAS institute Inc., Cary, NC, USA) using the PROC GLIMMIX procedure for a generalized linear mixed model. The farm was considered the experimental unit. The model included fixed effects of sample collection time point, location the sample was taken (well vs. rooms), and the interaction term between location (well vs. rooms) and the sample collection time point, with farm as a random effect. Post-hoc pairwise comparisons of least squared means were performed through Tukey’s adjustment for multiple comparisons. Total coliforms, TDS, iron, manganese, *E. coli*, and nitrate raw values were log-transformed using the natural base e. Total coliforms will be referenced throughout the results and discussion as coliforms or total coliforms. *P-*values of < 0.05 were considered significant.

Exploratory sensitivity analyses were performed for selected outcomes using available variables in the dataset. For log-transformed total coliform counts, additional models evaluated water temperature, on-farm pH, in-lab pH, water administered antimicrobial status, and frozen sample status as covariates. For log-transformed TDS, additional models evaluated water temperature and on-farm pH as covariates. For zinc, additional exploratory models evaluated on-farm pH, water temperature, water administered antimicrobial status, and hardness as covariates. These sensitivity analyses used the same general model structure as the primary analyses, including fixed effects for collection time point, sample location, and their interaction, with farm/site included as a random effect. These analyses were considered exploratory and were used to determine whether available covariates materially changed the primary conclusions.

## Results

Out of all assessed water quality parameters, few demonstrated significant differences or were approaching significance either between the location, across sample collection dates, or the interaction between location and sample collection date over the six wean-to-finish farms. Total coliforms, zinc, TDS, on-farm pH and in lab pH, and water temperature demonstrated significant differences (*P*-value ≤ 0.05). Average and ranges of water qualities from all farms and all parameters are recorded in Table 1.

**Table 1:** Water quality averages and ranges across all six wean-to-finish farms and water sample locations sampled in the study.

Water quality parameter	Average	Range
**Water hardness as CaCO_3_, ppm**	1171.6	334.1–1947.6
**Calcium, ppm**	294.3	92.4–449.5
**Copper^a^, ppm**	0.001	0–0.1
**Iron^a^, ppm**	0.558	0–7
**Magnesium, ppm**	106.0	23.3–201.5
**Manganese^a^, ppb**	1435.7	0–4185
**Molybdenum^a^, ppb**	3.2	0–6
**Phosphorus^a^, ppm**	N/A^b^	N/A^b^
**Potassium, ppm**	9.4	1.3–17.9
**Selenium^a^, ppb**	3.2	0–28
**Zinc^a^, ppm**	0.01	0–0.2
**Total coliform count per 100 mL^a^**	919.25	0–2419.6001
**Total *E. coli* per 100 mL**	169.31	0–2419.6001
**Nitrate, ppm**	11.49	0–111.3
**TDS, ppm**	2413.8	400.0–6700.0
**Sulfates, ppm**	1380.9	53.7–2370
**On-farm pH**	7.49	6.49–8.03
**In-lab pH**	7.37	6.68–8.02
**Water temperature °C**	19.8	12.6–29.8

a Indicates values with levels of quantification above zero. Values of affected results were adjusted to zero to be able to calculate averages.

b N/A; no values were reported above the level of quantification for this parameter over all samples.

ppm, parts per million; ppb, parts per billion.

### Coliforms

Coliforms had significant differences over sample collection dates (*P*-value = 0.0052), the location the sample was taken (*P*-value = 0.0024), and for the interaction between location and sample collection date (*P*-value ≤ 0.0001).

**Figure 1:**
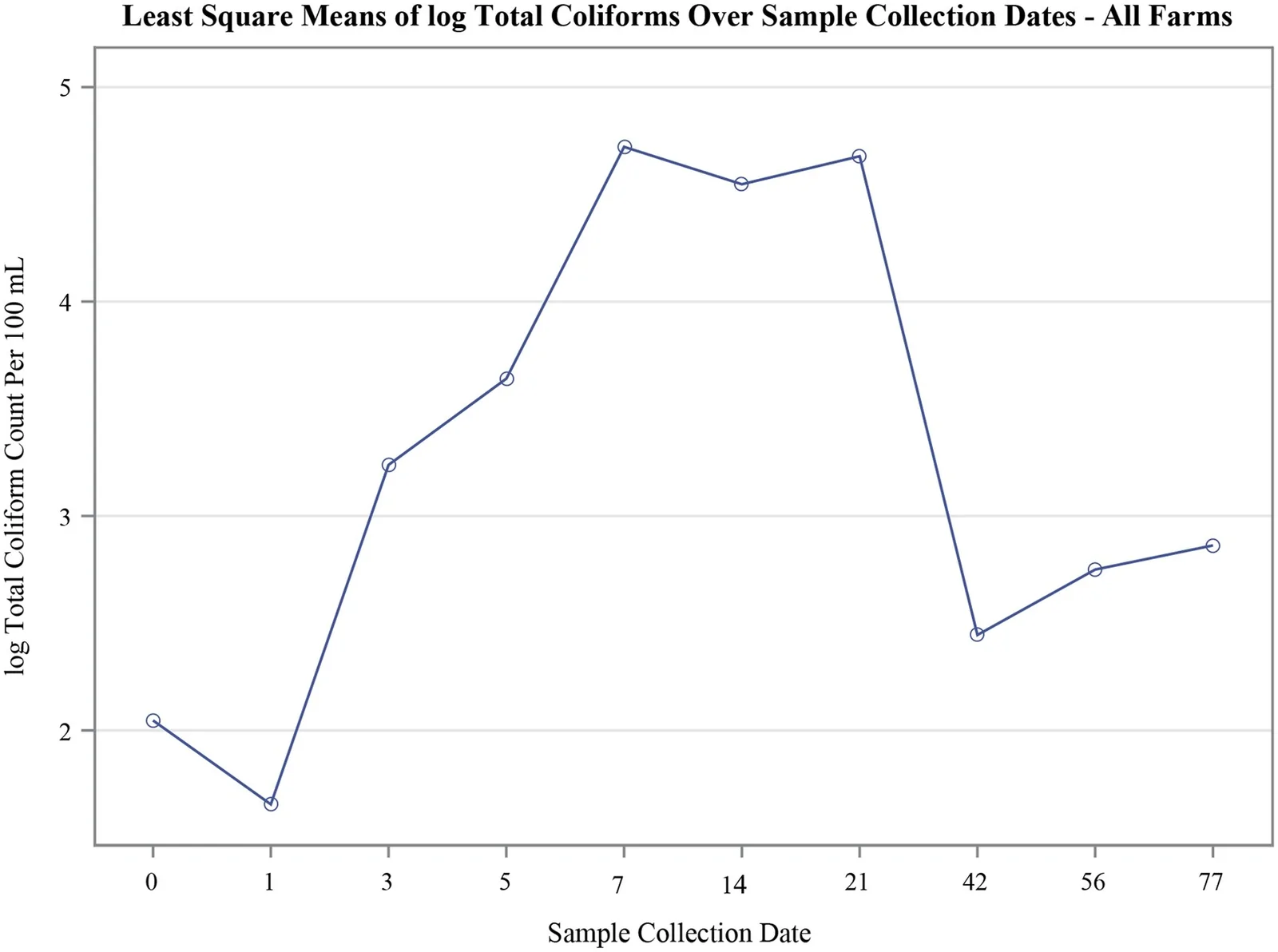
Least squares means for log total coliform count per 100 mL of water over the sample collection dates across all water sample locations and all six farms.

Post-hoc pairwise comparisons between sample collection dates demonstrated that there was no significant difference between pre-treatment (0) and post-treatment (1) with PAA (adjusted *P*-value = 1.0000). Significant differences at the 0.05 level were observed between the post-treatment sample collection date 1 and day 7 (adjusted *P*-value = 0.035) and between day 1 and day 21 (adjusted *P*-value (0.040). Coliforms were not statistically different between pre-treatment (0) and any post-treatment sample collection date. However, coliform estimates peaked at day 7 and remained elevated until sample collection day 21. Although not all pairwise comparisons were statistically significant after Tukey adjustment, coliform least-squares mean estimates increased numerically after treatment, particularly in room samples, and peaked around day 7 before declining. Figure 1 demonstrates the least squares means for the log of coliforms over all sample collection dates.

**Figure 2:**
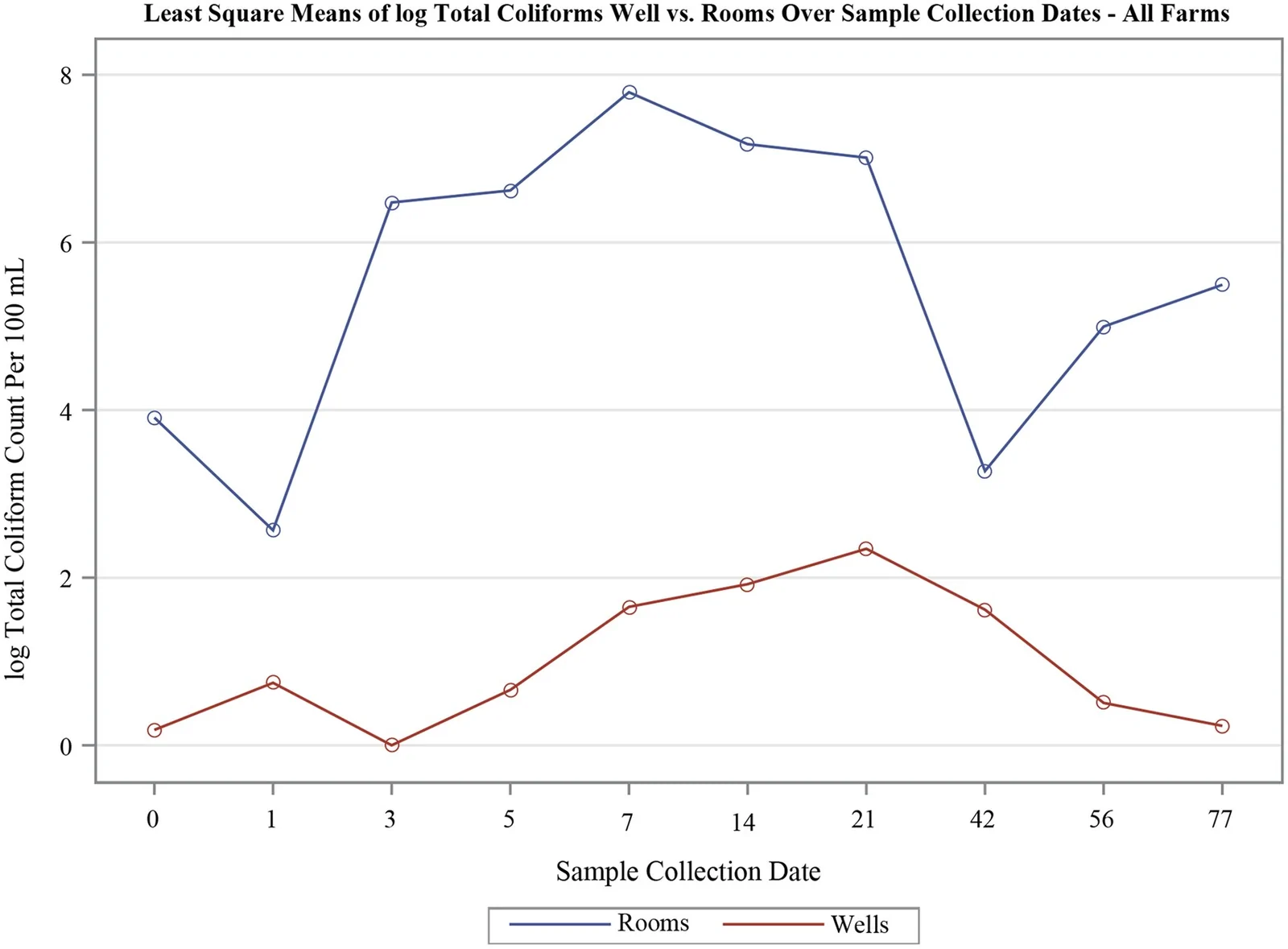
Least square means for log of total coliforms per 100 mL for all rooms vs. all wells over all sample collection dates. Water samples collected from rooms had significantly greater coliforms than water samples collected at the well (*P*-value = 0.0024). *P*-values of < 0.05 were considered significant.

Assessment of sample collection location demonstrated that coliform counts from well collected water samples were statistically different from coliform counts in water samples collected from the rooms of the farm (Fig. 2) (*P*-value = 0.0024). Coliforms were statistically greater in rooms than wells.

The interaction term of location and sample collection date were significant. When assessing the comparisons interaction at the level of the sample collection date, all but two collection time points were statistically significant with Tukey-Kramer’s adjustment for multiple comparisons (adjusted *P*-value ≤ 0.05). Post-treatment (1) and post-treatment day 42 were not statistically significant (adjusted *P*-value ≥ 0.05).

Sensitivity analyses were performed to evaluate whether measured water temperature, pH, antimicrobial status, or frozen sample status altered the primary coliform findings. The effects of collection time point, sample location, and the collection time point-by-location interaction remained significant for coliforms after adjustment for these variables. Water temperature, on-farm pH, in-lab pH, antimicrobial status, and frozen sample status were not statistically significant covariates in these models.

### Zinc

Zinc was statistically significant for the interaction between water sample location and sample collection date (*P*-value = 0.0196) and was approaching significance when assessed by sample collection date (*P*-value = 0.0541).

### Total dissolved solids

**Figure 3:**
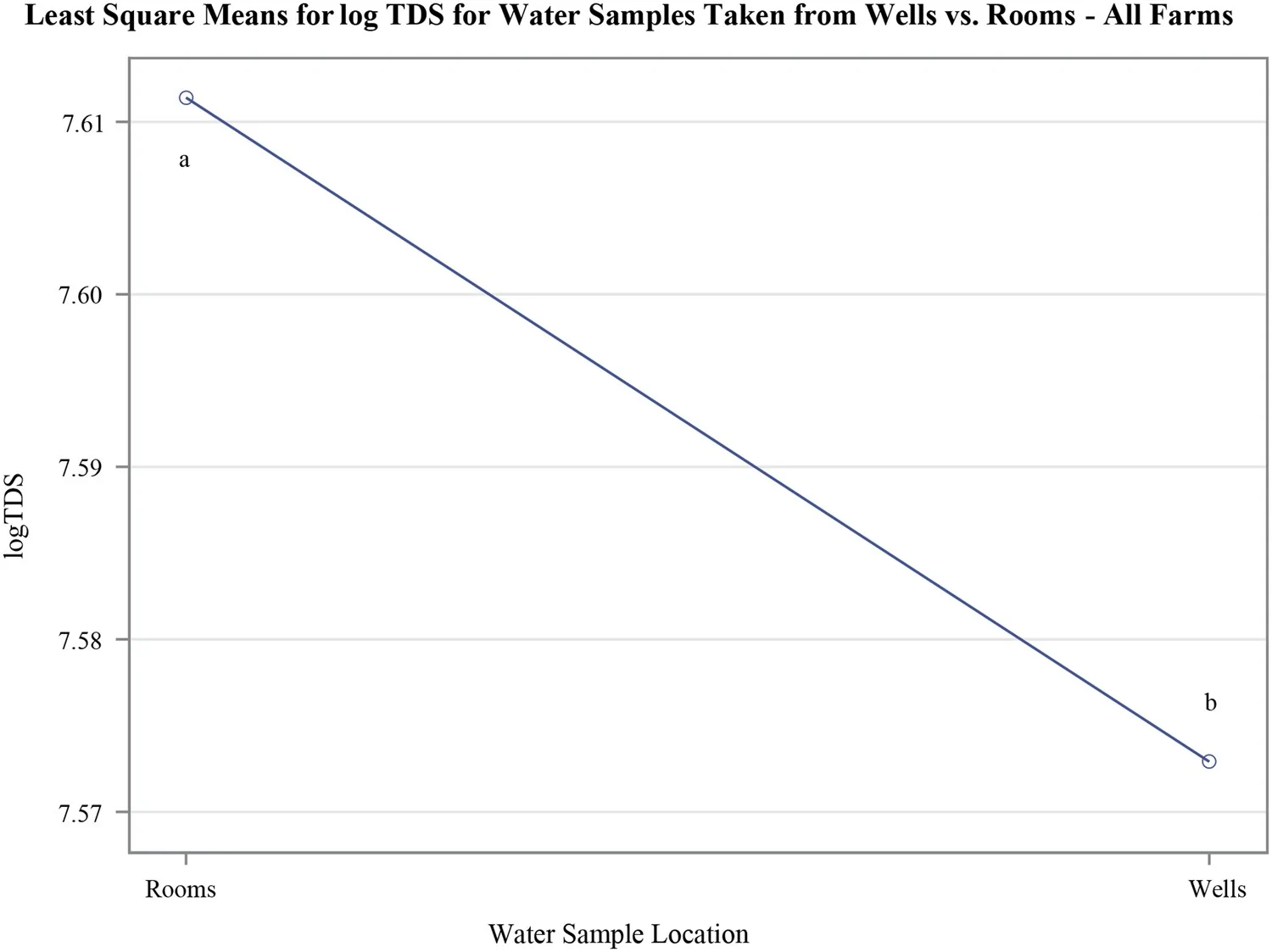
Least square means for log TDS (total dissolved solids) for water samples taken at rooms vs. wells for all farms. Room TDS values were significantly greater than TDS values from well water samples (*P*-value = 0.0323). Letters a and b indicate statistical significance (*P*-value < 0.05) between the water sample locations.

Total dissolved solids were statistically significant between water sample locations (*P*-value = 0.0323) as seen in Fig. 3, and for the interaction between water sample location and sample collection date (*P*-value = 0.0122). Room TDS estimates were greater than well estimates.

### On-farm and in-lab pH

**Figures 4–5:**
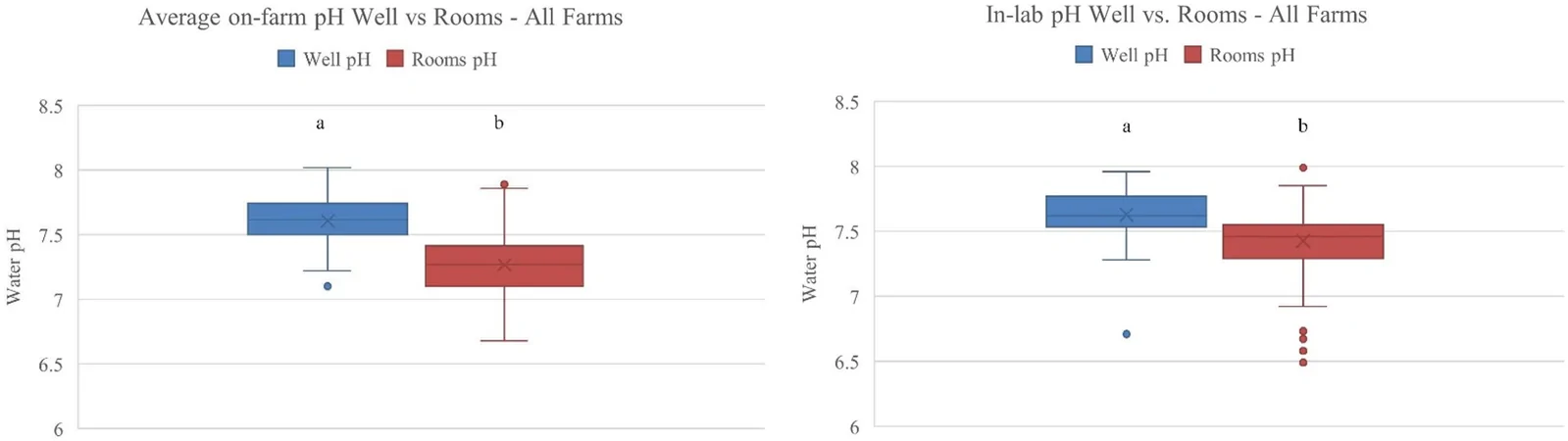
Water pH was measured immediately on-farm and again at the laboratory. Significant differences in water pH were noted for both (A) on-farm (*P*-value ≤ 0.0001) and (B) in-lab (*P*-value = 0.0002) pH values between the well and the rooms. Letters a and b indicate significance (*P*-value < 0.05) between the water sample locations.

On-farm pH was significantly different depending on the location the water sample was taken (*P*-value ≤ 0.0001), and the interaction term between water sample location and sample collection date (*P*-value = 0.0057). On-farm pH was significantly greater in the well samples versus in the rooms. In-lab pH demonstrated significant differences in the location the water sample was taken (*P*-value = 0.0002), as the pH was significantly greater in the well samples than the rooms (Figs 4-5), matching the on-farm results.

**Figure 6:**
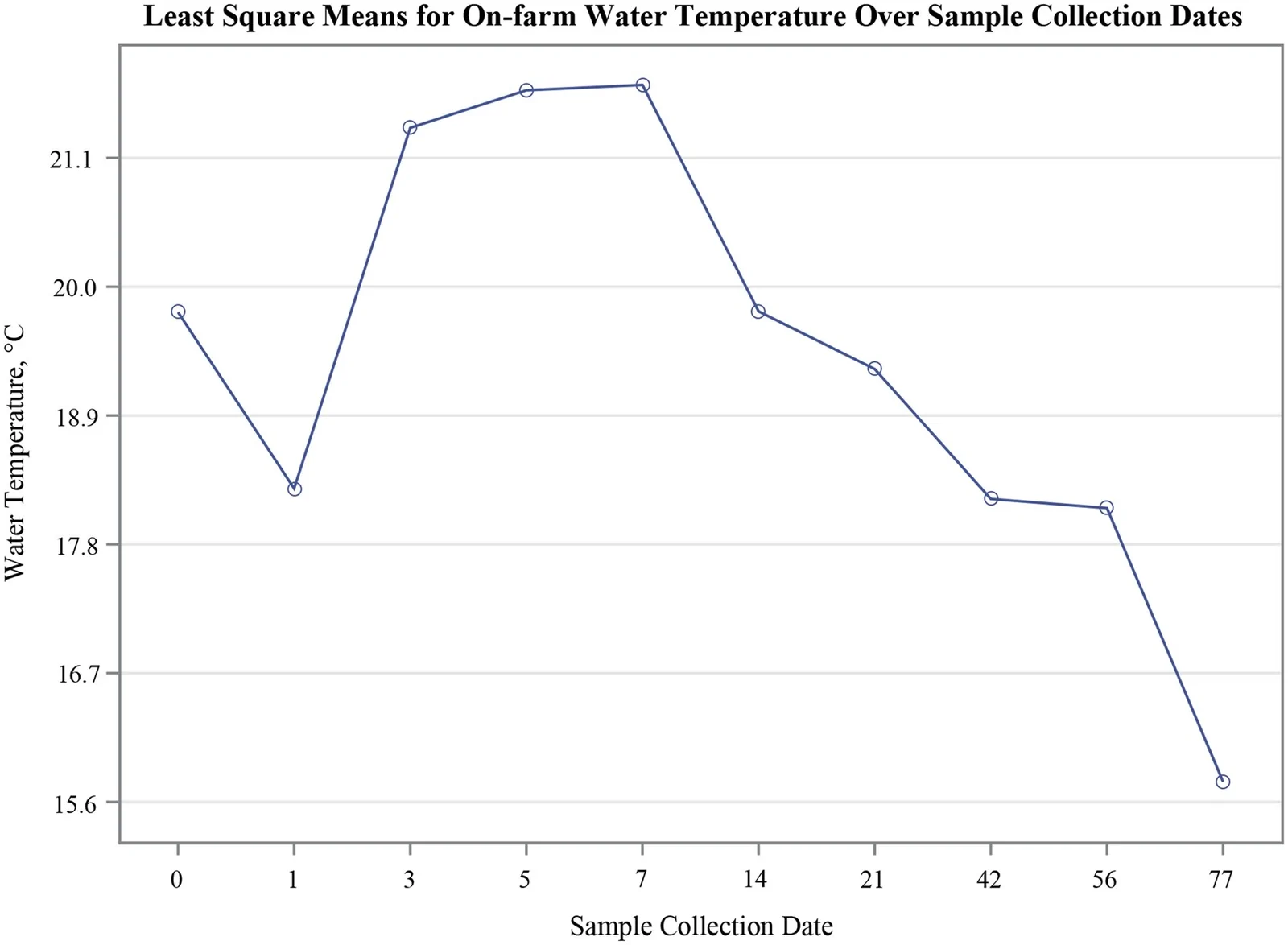
Least square means of water temperature taken on farm over sample collection dates from all water samples and farms. Water temperature demonstrated significant differences for sample collection date taken (*P*-value < 0.0001).

### Water temperature

**Figure 7:**
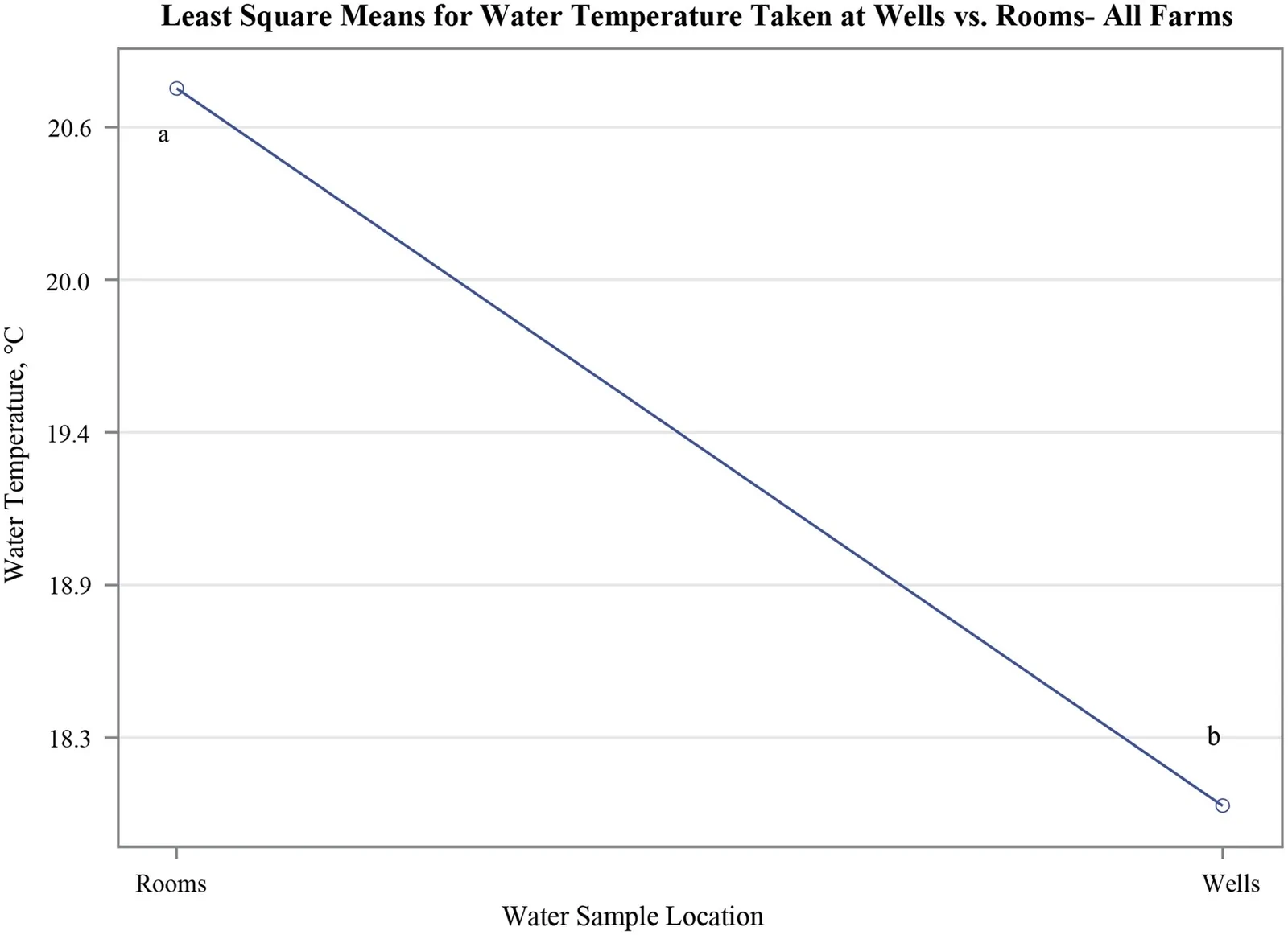
Least square means for water temperature taken at wells vs. rooms for all farms. Water temperature demonstrated significant differences for the location the water sample was taken with room water sample temperatures being greater than well water samples (*P*-value = 0.0391). Letters a and b indicate significance (*P* = <0.05) between the water sample locations.

Water temperature taken on-farm was statistically significant by sample collection date (*P*-value ≤ 0.0001) as seen in Fig. 6, water sample location (*P*-value = 0.0391) as seen in Fig. 7, and the interaction term between water sample location and sample collection date (*P*-value ≤ 0.0001). When the interaction term was assessed by sample collection date, all sample collection dates were statistically significant (adjusted *P*-value ≤ 0.05) except pre-treatment (0), immediately post-treatment (1), and day 77.

### Frozen, antimicrobial or electrolyte containing samples

Twelve water samples from four sample collection dates were frozen at −70 °C and thawed before submission. There were no statistical differences detected in chemical or microbial values from unfrozen compared to frozen samples (*P*-value = 1.000). The effect of water administered antimicrobials and electrolytes were assessed and no statistical differences in other water quality parameters were appreciated. Water administered antimicrobial status was evaluated as an exploratory sensitivity covariate for log-transformed total coliform counts. Water administered antimicrobial status was not statistically significant, and the main effects of sample location and the sample location-by-time interaction remained significant. No water samples demonstrated measurable oxidizing oxygen from any time points.

## Discussion

The study’s first null hypothesis could be partially rejected for a few of the water quality parameters assessed: coliforms, zinc, TDS, on-farm and in-lab pH measurements, and water temperature. The second null hypothesis that water quality is not significantly different between sampling locations was also only able to be partially rejected regarding coliforms, TDS, both pH measurements, and water temperature. Significant differences were observed for coliforms, zinc, TDS, on-farm and in-lab pH, and water temperature when assessed either for sampling location, over sample collection dates, or the interaction between location sample taken and sample collection date. These findings provide novel information about water quality dynamics in swine farms, offer insights into how water line cleaning and disinfection with 0.78% PAA can impact water quality, and provide additional guidance for water quality sampling.

Lack of statistical significance in most chemical parameters informs guidance for water sampling on swine farms. These results may suggest that minerals could be relatively stable over time and between sampling locations (rooms vs. well source) when multiple samples are collected on the same date. Therefore, one water sample may be representative for most mineral components for the entire farm, reducing resources directed at testing minerals for multiple samples. Alternatively, sampling water from one location alone for microbial assessment would not be representative of the entire farm based on the present study’s findings. Similar results showed that water collected at water drinkers was greater in coliforms than samples collected at the source (Edwards and Crabb 2021). This present study collected samples from main water lines instead of drinkers which likely had less environmental contamination as the sample location was not directly in contact with the pigs. In this present study, coliforms demonstrated statistical differences at the different sampling locations, indicating that multiple samples must be taken to be representative of the farm’s microbial water quality. Obtaining samples from both the well and room(s) are necessary to get comprehensive estimations of true drinking water quality for the pig.

Significant differences were not reported for most minerals over time across all farms. Zinc was the only mineral that demonstrated significant differences for the interaction between water sample location and sample collection date and was approaching significance for sample collection date. These results may suggest that influences on water quality are associated with sample collection date, for example, well water recharge and slight changes with mineral components in the aquifer over time could have influenced the results. Furthermore, it’s possible that zinc mobilized from the well’s galvanized steel piping could have contributed to the zinc levels reported in the study. Zn concentrations may be influenced by water temperature, hardness/mineral scale, plumbing materials, or other water chemistry factors. However, pipe material, mineral scale, and biofilm-associated metals were not directly measured, so mechanisms explaining Zn variation could not be determined.

This present study design may not have been sensitive enough to detect all biologically meaningful changes in water quality. In fact, on one site, visual inspection of the data suggested an event where some water quality parameters and mineral values (TDS, calcium, manganese, potassium, nitrates, and selenium) changed rapidly between sample collection days 3 and 5. Changes were maintained until day 14, then returned back to baseline values by sample collection day 21. These trends demonstrated that water quality can change within a matter of days. Influence from groundwater recharge could be a working hypothesis, however it is difficult to determine the definitive cause (Lindsey et al. 2023). Because of how the study was designed, over interpretation of results at an individual site level was avoided, and therefore this change in water quality parameters was likely under-represented in our analysis. Further research of temporal water parameter variability on swine farms needs to be investigated to determine the optimum intervals of sample collection for water monitoring and determine its relative importance to pig health.

### Total dissolved solids

Total dissolved solids were statistically significant based on the water sample location and the interaction between water sample location and the sample collection date. TDS are composed of multiple analytes that characterize the total inorganic matter of water or salinity of water (Carson 2000; NRC 2012). Analytes generally included in TDS include calcium, magnesium, sodium, bicarbonate, chloride, and sulfate (Carson 2000; Patience 2011; NRC 2012). Increases in some of these parameters would directly increase overall TDS values. It is possible that some parameters that were not directly measured in this study such as chlorine, bicarbonate, and sodium could have statistical trends, but were masked by other composite values in TDS. One hypothesis for these findings could include that soil particulates could have been separated out by in-line microfilters commonly found in swine farms, or perhaps there are influences in TDS due to biofilms, which could create differences based on water sample location. Water temperature was noted to be greater in rooms versus water sampled at the well and could have been influential on TDS solubility. This is an unlikely hypothesis as increases in other analytes would have been apparent, although it is possible the influences were in unmeasured components of TDS in this study. Residual mineral scale inside of original, main water lines within the farm could also have contributed to increased TDS levels. Future work is needed to explain the discrepancies in sampling location and the interaction term with sample collection date.

### Coliforms

Coliforms are used as indicators of potential fecal contamination but may or may not be useful in predicting the presence of pathogens (Richiardi et al. 2023). However, they can indicate potential livability conditions for pathogens with similar characteristics and other organisms in swine drinking water. Thus, their presence could represent a biosecurity risk on farms. This study found that water line cleaning and disinfection with 0.78% PAA did not statistically impact coliforms between pre- (0) and post-treatment (1) collection dates. The well and the water line pipe connecting the well to the main water distribution system before the water medicator to apply the PAA were not included in the water line cleaning and disinfection. This un-treated area of the water distribution system could have served as a reservoir for coliforms to enter the bulk water supply and seed the rest of the water distribution system for biofilm development. Any residual biofilm in water line pipes after the 0.78% PAA treatment could have also contributed to this finding. Other unmeasured factors may have contributed to coliform dynamics, including pig placement timing, retrograde contamination from drinkers or nipples, water usage or flow rate, recent water-administered products, and other environmental or management factors. These were not part of the original study design and could not be fully evaluated.

This study found that the presence of coliforms in the bulk water supply numerically increased above pre-treatment counts and peaked on sample collection d 7 and remained elevated until d 21. The numerical peak around d 7 could be consistent with a delayed biofilm regrowth or shedding process (Doughanet al. 2026). However, biofilm ecology was not directly measured in this study, and other unmeasured factors may also have contributed; therefore, this interpretation should be considered exploratory. Coliform regrowth after disinfection is a long-recognized issue in human water treatment systems (Dutka 1973; LeChevallier 1990; Kilb et al. 2003). However, these regrowth dynamics are poorly understood in livestock farms. Mechanisms to inactivate and prevent coliform and biofilm regrowth with continuous disinfection is a focus of modern human water treatment, which is not applicable to this current study conditions as only a one-time disinfection event was applied. However, much older research demonstrates the magnitude of coliform regrowth from treated wastewater effluent in tidal basin water and sewage effluent. These studies may represent similar water microbial conditions to a one-time disinfection process in swine farms (Shuval et al. 1973; Kinney et al. 1978). These studies demonstrated an increase in coliforms in the effluent after chlorine application, but longitudinal data past 7 d was limited. The study by Shuval (Shuval et al. 1973) demonstrated decline in coliform levels beginning at d 5, which was contrary to the present study’s findings of even longer elevated duration. They hypothesized this occurred due to potential nutrient availability after chlorination or the absence of competitive microorganisms (Shuval et al. 1973). This comparison is limited as study conditions may differ and both studies evaluated chlorinated wastewater instead of PAA. This comparison provides guidance and potential hypothesis to the occurrence of these dynamics in swine farms water systems and requires additional investigation.

In the water distribution system environment, there could be many contributing factors to the finding of coliform regrowth such as biofilm presence, temperature, nutrient availability, hydraulic effects, and others (LeChevallier 1990). An alternative hypothesis for the increased coliforms long-term post-treatment with PAA could also be explained by biofilm regrowth dynamics or characteristics of organisms in the biofilm (Doughanet al. 2026). Present water coliforms may be an indicator of mature biofilm organisms shedding into the bulk water supply, which describes the increase in coliforms over the sample collection dates. Bacteria can go into a viable but non-culturable state (VBNC), which is not metabolically active, but can revert to a metabolically active state in more opportune conditions (Li et al. 2014; Douterelo et al. 2016; Wang et al. 2021). This behavior of the bacteria in VBNC could potentially explain the results demonstrated in this study. Alternatively, another hypothesis could include that biofilm was incompletely removed during the PAA administration and secondary shedding into the bulk supply during the biofilm’s replication phase of regrowth occurred.

Additionally, there are a few factors that could have limited the coliform results. Coliforms were likely underestimated through the limitations of the Colilert IDEXX test. Colilert tests for enumerating coliforms has been accepted as a standard approach (Tambi et al. 2023) and is utilized frequently for quality control for water destined for human consumption. The Colilert test has an upper limit of detection of total coliforms up to 2419.6001 count per 100 mL. Several coliform results reached the upper reporting limit of the assay. Therefore, elevated coliform counts may have been underestimated, and differences among highly contaminated samples may have been compressed. Future studies should include serial dilutions or additional culture-based enumeration methods to better quantify samples exceeding the assay range. Furthermore, Colilert is an enzymatic method for enumeration of total coliforms and *E. coli* which requires the coliform to metabolize β-galactosidase and changes from clear water to yellow to indicate presence which are then enumerated through Quanti-Tray through fluorescence detection. Two samples collected on d 42 from one farm had yellow-colored antimicrobials in the water, which interfered with the interpretation of this test. Therefore, samples were reported as no detections, thus potentially impacting the results.

Although great care was taken to ensure cleanliness of sample collection, it is possible that potential field contamination from incomplete sanitation of the sample collection location (flush valve or spigot) could have contributed to reported coliforms. Additionally, twelve water samples were frozen due to the inability to submit the samples to the ISU VDL within 3 d after sample collection. When statistically tested, there were no significant differences in coliforms between frozen and unfrozen samples, but findings could be limited due to the small sample size. Furthermore, water was medicated with antimicrobials for 24 samples at 12 sample collection dates. Antimicrobials were only administered to the rooms and the well sample was not affected. Water antimicrobial administration was not randomized and occurred only in room samples. Therefore, water administered antimicrobial status could not be interpreted as an independent causal effect, and the analysis was used only as an exploratory sensitivity check. There were no statistical differences with assessed water quality parameters when antimicrobials were administered in the water, but this finding could have been affected by the limited sample size and the determination of how antimicrobials affect water quality was outside of the scope of this present study.

### On-farm vs. In-lab pH measurements

On-farm and in-lab pH measurements were evaluated as air exposure to samples between sample collection and submission to the laboratory could allow for carbon dioxide exchange with water and subsequent acidification of the sampled water through the creation of carbonic acid (Harned and Davis 1943). Differences in pH could have been appreciated between on-farm samples and in-lab taken samples for a few reasons. The first is potential changes in temperature from sample collection on farm to when it was tested in the lab which could have affected the solubility of carbon dioxide, and subsequently affecting the amount of carbonic acid created in water (Harned and Davis 1943). Additionally, methods for testing pH were different between in-lab and on-farm pH, thus potentially introducing variation in the values reported. As demonstrated by the total average of all water samples in the study, average in-lab pH was more acidic than on-farm pH, however the range of on-farm pH was wider, suggesting increased variability. The study objective was not to determine the best method for pH sample collection, but to account for large potential differences between on-farm and in-lab pH values. Both on-farm and in-lab pH values were similar, suggesting that either method could be utilized to measure water pH. Statistical differences were noted for both methods of pH measurement between the well water sample and the room collected water samples but remained within normal suggested limits for swine and were comparable to other study findings (Edwards 2018; Edwards and Crabb 2021; Münster and Kemper 2024). There are a few factors that could contribute to the significant differences in sampling location. Factors in temperature, carbon dioxide levels, and other water parameters could contribute to temporary changes in water quality from the well to the water lines in the farm adjusting the pH. Another hypothesis could include that the in-line filter (filters water from the well before entering water lines in the farm) could filter out macronutrients also adjusting pH between the sampling locations. Most minerals commonly measured in water were assessed and were not found to be statistically significant between sampling locations, therefore, lack of certain minerals in room water samples was unlikely to have affected the pH. The final possibility could include biofilm influence on decreased water pH inside the farm. Through biofilm metabolism and metabolism by-products, biofilms could affect pH. In dental biofilms, it has been demonstrated their metabolism can cause a decrease in environmental pH (Senneby et al. 2017), while sulfate reducing bacteria can produce hydrogen sulfide, which could also decrease pH (Barton and Fauque 2009). Complete understanding of this finding is limited as the level or composition of biofilm at the well was not assessed, which may or may not have been comparable to biofilms found in water lines. On-farm pH also was significantly different for the sample collection date. These findings highlight that there are additional factors that need to be investigated regarding water line ecology to comprehensively understand the influences of pH of swine drinking water on farms.

### Water temperature

The temperature of water sampled demonstrated significant differences at the sample collection date, the location the water was taken, and the interaction between the location the sample was taken and the sample collection date. The significant water temperature change at the sample collection date could have reflected the environmental conditions in the barn and the water consumption of the pigs. In between groups of pigs in a wean-to-finish farm, the temperature is often raised to facilitate drying of the facility and to prepare for the environmental needs of the incoming weaned pigs. Water pipes are adhered to the ceiling, exposed, and generally non-insulated. This could allow for warming from the farm environment. As the pigs grow over time, their environmental temperature requirements change and ambient temperatures decrease to maintain pig comfortability (Baker 2004).

Pigs increase their overall water consumption over time, which would impact on-farm water temperature. Groundwater is colder with temperatures estimated around 48 to 52 °F (8.9 to 11.1°C) (from northern to Southern Iowa, respectively) (Prior et al. 2003). When water consumption is low, water spends more time in the water distribution system and can be subject to warming before consumption. When water consumption increases, water spends a shorter amount of time in the water distribution system before being consumed by the pig and thus decreases water temperature. Visual differences between pre-treatment (0) and post-treatment (1) temperatures were apparent, where post-treatment (1) water samples were colder than pre-treatment on average. Water flowing through the water distribution system increased during this time, as the system was flushed to ensure proper application of PAA to all drinking water apparatuses. After the 24-h contact time with PAA, the water distribution system was flushed completely again to remove the PAA from the system. This water usage was the most likely contributor to decreased water temperatures for the post-treatment (1) sample collection date. Data of original on-farm environmental temperatures between groups of pigs were not collected in this study, and data for pig water consumption of all participating farms was not available for further assessment. However, water requirements and patterns of consumption in pigs have been well studied (Brumm 2006, 2010; Whittington and Gonyou 2007; NRC 2012; Patience 2013; Little et al. 2023). Understanding water temperature trends and dynamics in swine farms is important as it can potentially affect resident biofilm characterization (Douterelo et al. 2016; Revetta et al. 2016; Siedlecka et al. 2021), growth conditions (Siedlecka et al. 2021), or potentially biofilm shedding dynamics (Fish et al. 2016). These results should be considered for future water quality and water line ecology studies.

### Water quality averages across farms

In general, average water quality across the six farms demonstrated that all farms had hard water (increased calcium and magnesium concentrations) above 200 ppm with average on-farm and in-lab pH being alkaline. These water quality combinations could drive mineral scale on the inside of water lines (Carson 2000; Bahadori 2010). Based on previous literature, water hardness has not been demonstrated to have health or growth performance effects on pigs (Carson 2000; Patience 2011). Most of the other water quality parameters fell within acceptable guidelines and ranges considered for swine drinking water from previous literature (USEPA 1973; NRC 1974; Toft et al. 1987; Carson 2000; Patience 2011; Edwards 2018; Menegat et al. 2019; IPIC 2022). For some water quality analytes, such as iron and manganese, no nutrient limit has been generally established (Patience 2011). Iron and manganese may not have direct health effects to pigs, but iron can fuel bacteria growth and both minerals can cause discoloration of water line pipes and water nipples. Therefore, it has been recommended to keep these at minimal concentrations (Patience 2011; Edwards 2018). Others, such as sulfates, TDS, total coliforms, and *E. coli* had instances or were consistently considered outside of acceptable guidelines for pig consumption. Sulfates above 1000 ppm may cause osmotic diarrhea, although most studies suggest that growth performance and other health problems are not affected (Anderson and Stothers 1978; Paterson et al. 1979; DeWitt et al. 1987; Veenhuizen et al. 1992; Anderson et al. 1994; Carson 2000; Patience et al. 2004). Pigs can also adapt to water quality over time, so younger piglets, or new pigs that are introduced to a farm may be more affected by these water quality parameters (Patience 2011). Sulfates are also accounted for in TDS, and therefore TDS elevations were likely increased out of acceptable ranges due to their presence and the water hardness of the farms. Total dissolved solids over 3000 ppm have been recognized as another contributor for osmotic diarrhea but, in general, have also demonstrated not having any adverse health effects in pigs (Paterson et al. 1979; Ensley 1998; Carson 2000; NRC 2012; Samuel 2022). As described earlier in this paper, many acceptable ranges have been proposed for total coliforms in swine drinking water (USEPA 1973; Socha et al. 2003; NRC 2012; Edwards 2018; Menegat et al. 2019), and due to testing limitations in this study, the maximum levels of total coliforms reported with are uncertain. More research is needed to determine the outcomes of coliforms on health, performance, and biosecurity to determine the impact total coliforms and *E. coli* can have on pigs.

Although sulfates and nitrates tend to be well tolerated by pigs, it is worth noting that other species can be greatly affected by increased concentrations of these parameters in water. In this study, sulfate levels were elevated enough at some sites to be potentially lethal to cattle and could cause neurologic deficits as a result of polioencephalomalacia in both cattle and sheep (Carson 2000). No ruminants were associated with the well on the sampled farms. In ruminants, nitrates are converted to nitrites via rumen microbes, which eventually forms methemoglobin in the blood by which affected animals can demonstrate poor performance or weakness in younger animals (Undersander et al. 1999; Carson 2000). In contrast, pigs are monogastric animals and therefore don’t convert nitrates to nitrites and have demonstrated increased tolerance to greater levels of nitrates without health or growth performance effects (Anderson and Stothers 1978; Sorensen et al. 2021). It is also often forgotten that water should be considered an essential nutrient, and water quality is important to monitor as potential toxicities can occur with increased levels of different parameters (eg selenium) if compounded with what is present in the feed.

### Limitations

This study had several design limitations. The number of farms was limited, untreated control barns were not included, and all farms were not sampled simultaneously. Therefore, seasonal effects, precipitation patterns, barn environmental temperature, flow rate, water usage, and other management factors may have influenced the results. Water-administered medications may also have influenced microbial dynamics, but medication use was observational and not randomized. These factors should be considered in future controlled studies.

In this study, most of the common water quality parameters with available tests were collected to estimate differences in water quality between pre-treatment and post-treatment after administration of 0.78% PAA and to determine if there were variations in parameters between sampling locations (well and rooms). Other parameters could have been assessed and potentially demonstrated improvements after PAA administration or other trends between sampling locations. More work is needed in this area to complete water quality assessments on farms, especially when trying to improve water quality. For example, other parameters that could have been assessed were chloride, sodium, bicarbonate, arsenic, fluoride, lead, mercury, water conductivity, visual turbidity, pig palatability, and other measurements (Carson 2000; Patience 2013). Sodium and chloride were not selected for assessment as they were not included as part of the standard mineral panel available at the diagnostic laboratory. Unexplained values in TDS could have supported additional testing for sodium and chloride. However, no large differences or unexplained values could be appreciated in the study’s TDS that couldn’t be generally attributed to contributions from calcium, magnesium, and sulfates.

Additional limitations in sample collection could have occurred. Water samples contained 500 mL of water with 100 mL going to test total coliforms and *E. coli*, leaving 400 mL of water to test all other parameters. This is a very small representation of the greater water quality volume in the farm, which could be more variable. Based on the equipment used, many water quality parameters also have levels of quantification that are limited (eg <2, < 0.1 ppm, etc.). This directly affected our results as samples could have had 1 or 0.05 ppm but would not have met the threshold for quantification. This directly affected water quality averages calculated from all six farms. Finally, the PAA used was administered between groups of pigs at an off-label dose (labeled at 2% or 1:50), to accommodate common water medicators on swine farms of a 1:128 oz ratio or 0.78%. Water quality parameters may have improved or achieved different outcomes if the PAA administered at the 2% labeled dose.

Water quality is essential to maintaining pig health, biosecurity and productivity. Unique considerations of resident water quality, and potential microbial contamination either from the water source or from factors inside of the farm, can further exacerbate poor water quality on swine farms. This study evaluated if chemical and microbial water quality analytes would improve after water line cleaning and disinfection with 0.78% PAA and detect differences in water quality based on water sample location. Results found that room water quality, regarding its microbiological parameters, is significantly different than water samples collected at the well, and that 0.78% PAA did not significantly alter measured parameters between pre-and post-treatment sample collections. No residual water line disinfection was performed. Over time, total coliform estimates numerically increased and peaked at 7 d post-treatment with 0.78% PAA. This is potentially a representation of biofilm regrowth shedding into the bulk water supply. Water pH, TDS, and water temperature displayed significant differences between water sampling locations (well vs. rooms). Water temperature demonstrated significant differences over sample collection dates, and temperature decreased over time. Minerals remained relatively stable across sampling locations and were not significantly different over time. However, zinc was significantly different at the interaction between sample collection dates and water sample location. This study elucidates best practices for water line management and water sampling in swine farms.

## Data Availability

The datasets generated and/or analyzed during the current study are available in the Iowa State University Open Data repository “DataShare” at this link: https://doi.org/10.25380/iastate.29508191.v1.

## List of abbreviations

TDS: total dissolved solids
PAA: peracetic acid
TOO: total oxidizing oxygen
PPM: parts per million
PPB: parts per billion

## References

[txag110-B1] Anderson D. , Stothers S. 1978. Effects of saline water high in sulfates, chlorides and nitrates on the performance of young weanling pigs. J. Anim. Sci. 47:900–907. 10.2527/jas1978.474900x 738975

[txag110-B2] Anderson J. S. , Anderson D. M., Murphy J. M. 1994. The effect of water quality on nutrient availability for grower/finisher pigs. Can. J. Anim. Sci. 74:141–148. 10.4141/cjas94-021

[txag110-B3] Bahadori A. 2010. Prediction of scale formation in calcium carbonate aqueous phase for water treatment and distribution systems. Water Q. Res. J. 45:379–389. 10.2166/wqrj.2010.038

[txag110-B4] Baker J. 2004. Effective environmental temperature. JSHAP. 12:140–143. 10.54846/jshap/391

[txag110-B5] Barton L. L. , Fauque G. D. 2009. Chapter 2: biochemistry, physiology and biotechnology of sulfate‐reducing bacteria. In: A. I. Laskin, G. M. Gadd, and S. Sarislani, editors. Advances in applied microbiology no. 68. Academic Press, San Diego, CA. p. 41–98. 10.1016/S0065-2164(09)01202-7 19426853

[txag110-B6] Böger R. , Rohn K., Kemper N., Schulz J. 2020. Sodium hypochlorite treatment: the impact on bacteria and endotoxin concentrations in drinking water pipes of a pig nursery. Agriculture. 10:86. 10.3390/agriculture10030086

[txag110-B7] Boyd C. E. , Tucker C. S., Somridhivej B. 2016. Alkalinity and hardness: critical but elusive concepts in aquaculture. J. World Aquaculture Soc. 47:6–41. 10.1111/jwas.12241

[txag110-B8] Brumm M. 2006. Patterns of drinking water use in pork production facilities. University of Nebraska Lincoln, Nebraska Swine Reports, Lincoln, NE.

[txag110-B9] Brumm M. 2010. Water recommendations and systems for swine. https://porkgateway.org/resource/water-recommendations-and-systems-for-swine/

[txag110-B10] Carson T. L. 2000. Current knowledge of water quality and safety for livestock. Vet. Clin. North Am. Food Anim. Pract. 16:455–464. 10.1016/s0749-0720(15)30080-3 11084986

[txag110-B11] DeWitt P. , Young L., Wenzell R., Friendship R., Peer D. 1987. Water quality and pig performance. Can. J. Anim. Sci. 67:1196.

[txag110-B12] Dewulf , Immerseel J. F., Postma M., Rooster H., Damiaans B., Sarrazin S., Abma E., Maes D. 2018. Biosecurity in animal production and veterinary medicine. ACCO, New York, NY.

[txag110-B13] Doughan G. 2025. Characterization of the impacts of water line cleaning and disinfection on swine wean-to-finish water line ecology. Iowa State University, Iowa State University Digital Repository, Ames, IA.

[txag110-B14] Doughan , Karriker G. L., Mou K. 2022. Water biology: implications for pig health, production and biosecurity. In: Proceedings of the ISU James D. McKean Swine Disease Conference. Ames, IA. p. 66–71.

[txag110-B15] Doughan , Karriker G. L., Mou K. 2023. Water biology: the next frontier for biosecurity. In: Proceedings of the 54th Annual meeting of the American Association of Swine Veterinarians. Aurora, CO. p. 346–350.

[txag110-B16] Doughan G. et al 2026. Water line biofilm regrowth dynamics in six wean-to-finish farms post peracetic acid water line cleaning and disinfection. Sci. Rep. 16:9921. 10.1038/s41598-026-40725-x PMC13018522 41714774

[txag110-B17] Douterelo , Husband I. S., Loza V., Boxall J. 2016. Dynamics of biofilm regrowth in drinking water distribution systems. Appl. Environ. Microbiol. 82:4155–4168. 10.1128/AEM.00109-16 PMC4959196 27208119

[txag110-B18] Dutka B. J. 1973. Coliforms are an inadequate index of water quality. J. Environ. Health. 36:39–46.

[txag110-B19] Edwards L. 2018. Drinking water quality and its impact on the health and performance of pigs. Innovation Project 2A-118, Co-operative Research Centre for High Integrity Australian Pork, Canberra, Australia.

[txag110-B20] Edwards L , Crabb H. 2021. Water quality and management in the Australian pig industry. Anim. Prod. Sci. 61:637–644. 10.1071/AN20484

[txag110-B21] Ensley S. M. 1998. Relationships of swine water quality to cost and efficiency of swine production. Iowa State University, Iowa State University Digital Repository, Ames, IA.

[txag110-B22] Farjami A. , Hatami M. S., Siahi‐Shadbad M. R., Lotfipour F. 2022. Peracetic acid activity on biofilm formed by *Escherichia coli* isolated from an industrial water system. Lett. Appl. Microbiol. 74:613–621. 10.1111/lam.13647 34984695

[txag110-B23] Fish K. E. , Osborn A. M., Boxall J. 2016. Characterising and understanding the impact of microbial biofilms and the extracellular polymeric substance (EPS) matrix in drinking water distribution systems. Environ. Sci. Water Res. Technol. 2:614–630. 10.1039/C6EW00039H

[txag110-B24] Flemming H.-C. , Percival S., Walker J. 2002. Contamination potential of biofilms in water distribution systems. Water Sci. Technol. Water Supply. 2:271–280. 10.2166/ws.2002.0032

[txag110-B25] Harned H. S. , Davis R. Jr. 1943. The ionization constant of carbonic acid in water and the solubility of carbon dioxide in water and aqueous salt solutions from 0 to 50. J. Am. Chem. Soc. 65:2030–2037. 10.1021/ja01250a059

[txag110-B26] Iowa Pork Industry Center 2022. Water quality in swine barns: how do we define it? Iowa State University Extension and Outreach Extension Store. p. 1–3. https://shop.iastate.edu/extension/farm-environment/animals-and-livestock/swine/ipic204.html

[txag110-B27] Kampf G. 2024. Peracetic acid, antiseptic stewardship: biocide resistance and clinical implications. Springer International Publishing, Cham. p. 117–173.

[txag110-B28] Kilb B. , Lange B., Schaule G., Flemming H.-C., Wingender J. 2003. Contamination of drinking water by coliforms from biofilms grown on rubber-coated valves. Int. J. Hyg. Environ. Health. 206:563–573. 10.1078/1438-4639-00258 14626903

[txag110-B29] Kinney E. , Drummond D., Hanes N. 1978. Effects of chlorination on differentiated coliform groups. J. Water Pollut. Control Fed. 50:2307–2312. https://www.jstor.org/stable/25040152

[txag110-B30] Kitis M. 2004. Disinfection of wastewater with peracetic acid: a review. Environ. Int. 30:47–55. 10.1016/S0160-4120(03)00147-8 14664864

[txag110-B31] LeChevallier M. W. 1990. Coliform regrowth in drinking water: a review. J. Am. Water Works Assoc. 82:74–86. 10.1002/j.1551-8833.1990.tb07054.x

[txag110-B32] Li L. , Mendis N., Trigui H., Oliver J. D., Faucher S. P. 2014. The importance of the viable but non-culturable state in human bacterial pathogens. Front. Microbiol. 5:258. 10.3389/fmicb.2014.00258 PMC4040921 24917854

[txag110-B33] Lindsey B. D. , Fleming B. J., Goodling P. J., Dondero A. M. 2023. Thirty years of regional groundwater-quality trend studies in the United States: major findings and lessons learned. J. Hydrol. (Amst). 627:130427. 10.1016/j.jhydrol.2023.130427

[txag110-B34] Little S. , Woodward A., Browning G., Billman-Jacobe H. 2021. Water distribution systems in pig farm buildings: critical elements of design and management. Animals. (Basel). 11:3268. 10.3390/ani11113268 PMC8614494 34828000

[txag110-B35] Little S. B. , Woodward A. P., Browning G. F., Billman-Jacobe H. 2023. Water use patterns within each day: variation between batches of growing pigs in commercial production systems. JSHAP. 31:20–28. 10.54846/jshap/1297

[txag110-B36] Lozinski B. M. 2020. The effects of water quality on nursery pig performance and health [thesis]. Minneapolis, MN: University of Minnesota. https://www.proquest.com/docview/2488681198/84EB53ECD9444682PQ/1?accountid=10906&sourcetype=Dissertations%20&%20Theses

[txag110-B37] Lozinski B. M. et al 2022. Effects of water quality on growth performance and health of nursery pigs. Transl. Anim. Sci. 6:txac002. 10.1093/tas/txac002 PMC8827558 35155995

[txag110-B38] McAllister T. A. , Topp E. 2012. Role of livestock in microbiological contamination of water: commonly the blame, but not always the source. Anim. Front. 2:17–27. 10.2527/af.2012-0039

[txag110-B39] McLeese J. M. , Tremblay M. L., Patience J. F., Christison G. I. 1992. Water intake patterns in the weanling pig: effect of water quality, antibiotics and probiotics. Anim. Sci. 54:135–142. 10.1017/S0003356100020651

[txag110-B40] Menegat , Goodband M. R., DeRouchey J., Tokach M., Woodworth J., Dritz S. 2019. Water in swine nutrition. Kansas State University. p. 1–3. https://www.asi.k-state.edu/extension/swine/swinenutritionguide/general_nutrition_principles/water.html

[txag110-B41] Münster P. , Kemper N. 2024. Long-term analysis of drinking water quality in poultry and pig farms in Northwest Germany. Front. Anim. Sci. 5:1467287. 10.3389/fanim.2024.1467287

[txag110-B42] NRC. 1974. Nutrients and toxic substances in water for livestock and poultry: a report. NRC, Washington, DC.

[txag110-B43] NRC. 2012. Water, nutrient requirements of swine. Washington, DC: National Academies Press. p. 66–73.

[txag110-B44] Nyachoti C. , Patience J., Seddon I. 2005. Effect of water source (ground versus surface) and treatment on nursery pig performance. Can. J. Anim. Sci. 85:405–407. 10.4141/A04-086

[txag110-B45] Nyachoti M , Kiarie E. 2010 Feb 3–4. Water in swine production: a review of its significance and conservation strategies. In: Proceedings of the 24th Manitoba Swine Seminar; Winnipeg, Manitoba, Canada. p. 217–232.

[txag110-B46] Olsen P. C. S. , Steven J. 2020. Groundwater & livestock production and husbandry, part 1. In: Biosecurity 51st Annual Meeting of the American Association of Swine Veterinarians. AASV, Atlanta, GA. p. 384–398.

[txag110-B47] Paruch A. M. , Mæhlum T. 2012. Specific features of *Escherichia coli* that distinguish it from coliform and thermotolerant coliform bacteria and define it as the most accurate indicator of faecal contamination in the environment. Ecol. Indic. 23:140–142. 10.1016/j.ecolind.2012.03.026

[txag110-B48] Paterson D. W. , Wahlstrom R. C., Libal G. W., Olson O. E. 1979. Effects of sulfate in water on swine reproduction and young pig performance. J. Anim. Sci. 49:664–667. 10.2527/jas1979.493664x 528427

[txag110-B49] Patience J. 2011. Water quality issues in pork production. In: Proceedings of the Allen D. Leman Swine Conference; St. Paul, Minnesota. p. 157–164.

[txag110-B50] Patience J. F. 2013. Water in swine nutrition. In: Chiba L. I., editor, Sustainable swine nutrition. John Wiley& Sons, Hoboken, NJ.

[txag110-B51] Patience J. F. , Beaulieu A. D., Gillis D. A. 2004. The impact of groundwater high in sulfates on the growth performance, nutrient utilization, and tissue mineral levels of pigs housed under commercial conditions. JSHAP. 12:228–236. 10.1002/9781119583998.ch2

[txag110-B52] Prior , Boekhoff J. C. J. L., Howes M. R., Libra R. D., VanDorpe P. E., Vonk J. R. 2003. Iowa’s groundwater basics a geological guide to the occurence, use, &vulnerability of Iowa’s aquifers. In: Iowa geological survey educational series. Iowa Department of Natural Resources, Ames, IA. p. 83.

[txag110-B53] Revetta R. , Gomez‐Alvarez V., Gerke T., Santo Domingo J., Ashbolt N. 2016. Changes in bacterial composition of biofilm in a metropolitan drinking water distribution system. J. Appl. Microbiol. 121:294–305. 10.1111/jam.13150 27037969

[txag110-B54] Richiardi L. , Pignata C., Fea E., Bonetta S., Carraro E. 2023. Are indicator microorganisms predictive of pathogens in water? Water. (Basel). 15:2964. 10.3390/w15162964

[txag110-B55] Samuel R. 2022. Total dissolved solids (TDS) less than 1000 ppm in drinking water did not impact nursery pig performance. Vet. Sci. 9:622. 10.3390/vetsci9110622 PMC9695767 36356099

[txag110-B56] Senneby A. , Davies J. R., Svensäter G., Neilands J. 2017. Acid tolerance properties of dental biofilms in vivo. BMC Microbiol. 17:165. 10.1186/s12866-017-1074-7 PMC5525231 28743239

[txag110-B57] Shuval H. I. , Cohen J., Kolodney R. 1973. Regrowth of coliforms and fecal coliforms in chlorinated wastewater effluent. Water Res. 7:537–546. 10.1016/0043-1354(73)90053-5

[txag110-B58] Siedlecka A. , Wolf-Baca M., Piekarska K. 2021. Microbial communities of biofilms developed in a chlorinated drinking water distribution system: a field study of antibiotic resistance and biodiversity. Sci. Total Environ. 774:145113. 10.1016/j.scitotenv.2021.145113 33610999

[txag110-B59] Socha , Ensley M. T. S., Tomlinson D. J., Johnson A. B. 2003. Variability of water composition and potential impact on animal performance. In: Proceedings from the Intermountain Nutrition Conference; Salt Lake City, UT. p. 85–96.

[txag110-B60] Sorensen J. P. R. et al 2021. Seasonality of enteric viruses in groundwater-derived public water sources. Water Res. 207:117813. 10.1016/j.watres.2021.117813 34785409

[txag110-B61] Stull C. L. et al 1999. Assessment of bacteria and mycotoxins in feed and coliforms in water offered to high and low performing commercial growing hogs in California. Prof. Anim. Sci. 15:94–99. 10.15232/S1080-7446(15)31735-6

[txag110-B62] Tambi A. , Brighu U., Gupta A. B. 2023. Methods for detection and enumeration of coliforms in drinking water: a review. Water Supply. 23:4047–4058. 10.2166/ws.2023.247

[txag110-B63] Toft , Malaiyandi P. M., Hickman J. R. 1987. Chapter 35: Guidelines for Canadian drinking water quality. In: I. H. (Mel) Suffet and M. Malaiyandi, editors. Organic pollutants in water Sampling, Analysis, and Toxicity Testing. Vol. 214 . American Chemical Society. 10.1021/ba-1987-0214

[txag110-B64] Undersander , Combs D. D., Howard T., Shaver R., Siemens M., Thomas D. 1999. Nitrate poisoning in cattle, sheep and goats. University of Wisconsin-Madison Extension Cooperative Service, University of Wisconsin-Madison, Madison, WI.

[txag110-B65] USEPA. 1973. Proposed criteria for water quality: quality of water for livestock No. 4. Washington, DC: U.S. Environmental Protection Agency. p. 663.

[txag110-B66] Veenhuizen M. F. , Shurson G. C., Kohler E. M. 1992. Effect of concentration and source of sulfate on nursery pig performance and health. JAVMA. 201:1203–1208. 10.2460/javma.1992.201.08.1203 1429159

[txag110-B67] Walthart B. K. 2024. Optimizing swine water line management: a study of hydration status during water medication events and best practices for water line antimicrobial use. Iowa State University, Iowa State University Digital Repository, Ames, IA.

[txag110-B68] Wang M. , Ateia M., Awfa D., Yoshimura C. 2021. Regrowth of bacteria after light-based disinfection–what we know and where we go from here. Chemosphere. 268:128850. 10.1016/j.chemosphere.2020.128850 33187648

[txag110-B69] Whittington D. L. , Gonyou H. 2007. Water: production, performance and behavior considerations. American Association of Swine Veterinarians, Orlando, FL. p. 5–9.

